# Impact of subsidized fortified wheat on anaemia in pregnant Indian women

**DOI:** 10.1111/mcn.12669

**Published:** 2018-09-04

**Authors:** Suman Chakrabarti, Avinash Kishore, Kalyani Raghunathan, Samuel P. Scott

**Affiliations:** ^1^ Poverty Health and Nutrition Division International Food Policy Research Institute New Delhi India; ^2^ South Asia Office International Food Policy Research Institute New Delhi India; ^3^ Poverty Health and Nutrition Division International Food Policy Research Institute Washington, D.C.

## Abstract

The World Health Assembly called for a 50% global anaemia reduction in women of reproductive age (15–49 years of age) from 2012 to 2025. India accounts for the most cases of anaemia in the world, and half of all pregnant Indian women are anaemic. In India, the government implemented a 4‐year food‐based safety net programme from 2008 to 2012 involving the provision of fortified wheat flour through its public distribution system. We assessed programme impact on anaemia among pregnant women (*n* = 10,186) using data from the 2002–2004 and 2012–2013 Indian District Level Health Surveys. The difference‐in‐differences method was used to estimate the impact on haemoglobin (Hb) and anaemia in pregnant women living in northern India (Punjab) and southern India (Tamil Nadu), with pregnant women in neighbouring states without wheat fortification programmes serving as controls. In northern India, we found no impact on Hb (β = −0.184, *P* = 0.793) or anaemia reduction (β = −0.01, *P* = 0.859), as expected, given that the intervention targeted only nonpoor households and demand for fortified wheat was low. In southern India, where intervention coverage was high, we found no impact on Hb (β = −0.001, *P* = 0.998) but did see an impact on anaemia reduction (β = −0.08, *P* = 0.042), which was unexpected given low consumption of wheat in this predominantly rice‐eating region. India's wheat fortification programmes were largely ineffective in terms of reducing anaemia among pregnant women. As policymakers expand fortification programs, it is critical to ensure that the fortified food is universally available and distributed widely through well‐functioning and popular outlets.

**Key messages**

- Half of pregnant women in India are anaemic. Fortification of wheat distributed through India's public distribution system has the potential to reach a large percentage of the anaemic population in the country, but evidence on the effectiveness of this strategy is scarce.
- Using rigorous econometric methods, we conducted an impact assessment of a 4‐year government‐led effort to distribute fortified wheat in two Indian states on anaemia reduction among pregnant women. We found no impact in Punjab, where the distribution channel was dysfunctional, and a small impact in Tamil Nadu, where coverage was adequate but wheat consumption was low.
- Governments intending to use fortification as a strategy to combat anaemia should be mindful that programme effectiveness depends on regular consumption of the fortified food in adequate quantities by the target population, and distribution through popular outlets.

**List of abbreviations**
AP: Andhra Pradesh
APL: above poverty line
BPL: below poverty line
DID: difference in differences
DLHS: district level household survey
Hb: haemoglobin
HR: Haryana
IFA: iron folic acid
INR: Indian rupee
KA: Karnataka
KE: Kerala
NFHS: National Family Health Survey
NSS‐CES: National Sample Survey of Consumer Expenditure
OBC: other backward classes
PDS: public distribution system
PN: Punjab
TN: Tamil Nadu
WHO: World Health Organization

## 1 INTRODUCTION

Approximately one third of the global population is estimated to be anaemic (Lopez, Cacoub, Macdougall, & Peyrin‐Biroulet, 2016). This severe public health issue is especially problematic in women of childbearing age, and the second World Health Assembly global nutrition target is to halve anaemia in this group by 2025 (World Health Organization, 2015). Worldwide, 29% of nonpregnant women and 38% of pregnant women were affected in 2011 (World Health Organization, 2011b) and regionally, anaemia prevalence is highest in Southeast Asia (Stevens et al., 2013). Within South Asia, the most cases of anaemia occur in India. Half of pregnant Indian women are anaemic (Hb < 110 g/L) according to a recent national survey (Government of India, 2017), which is troubling, given that anaemia during pregnancy is associated with higher risk of low birth weight, preterm birth, and perinatal mortality (Rahman et al., 2016), as well as a higher risk of maternal mortality (Daru et al., 2018). The aetiology of anaemia is complex and context specific, though iron deficiency is a causal factor in about one quarter to one half of all cases, with other causes including haemoglobin disorders, other micronutrient deficiencies such as vitamin B12 and folate, blood loss through heavy menstruation or trauma, and disease states (Bahizire et al., 2017; Kassebaum et al., 2014; Petry et al., 2016; Wieringa et al., 2016). Among populations in developing countries with predominantly plant‐based diets such as India, poor iron status is typically due to low intake of bioavailable iron combined with chronic infection hindering iron absorption.

Iron supplementation is the fastest method of addressing clinical iron deficiency anaemia, but this strategy faces barriers, including low compliance due to gastric side effects; a risk of toxicity and exacerbation of concomitant infection (Sazawal et al., 2006; World Health Organization, 2007); and supply side issues stemming from poorly functioning health systems (Kosec et al., 2015; Maity, 2016). Alternatives to iron supplementation include promotion of dietary behaviour change to include more diverse and iron‐rich foods, biofortification of staple food crops using plant‐breeding methods, point‐of‐use fortification where powderized iron and other micronutrients can be sprinkled on food at the household level, and mass commercial fortification. Commercial fortification involves the addition of iron during the manufacturing or processing stage; is relatively cost effective (Menon, McDonald, & Chakrabarti, 2016); has little adverse health effects assuming appropriate regulation of fortificant levels; and has wide reach, as it targets all consumers of a particular food commodity.

Wheat flour fortification started in the United States in the 1940s and is practiced in approximately 78 countries (Hurrell et al., 2010). In 2000, West Bengal was the first state in India to fortify wheat flour, and now, an estimated 7.6% of industrially milled wheat flour in India is fortified (Food Fortification Initiative, 2017). There is currently momentum to expand flour fortification in India, and in October 2016, the Food Safety Standards and Authority of India published a draft of national guidelines (Food Safety and Standards Authority of India, 2016), in accordance with WHO interim guidelines from 2009 (World Health Organization, 2009), on safe and effective levels of iron, folic acid, and vitamin B12 to be added during wheat milling.

After the enactment of the National Food Security Act in 2013, the Indian Government scaled up coverage of the public distribution system (PDS) to reach approximately 66% of India's population with a per beneficiary quota of 5 kg of cereal, effectively making the PDS the largest food safety net in the world (Kishore & Chakrabarti, 2015). Beneficiaries were identified based on their household socio‐economic status. Those having a household income below a certain limit were issued Antyodaya Anna Yojana—“poorest of the poor”—or below poverty line (BPL) ration cards by the Department of Civil Supplies and Consumer Affairs and were eligible for benefits. In addition, certain special categories of households—for example, households that are homeless or living in temporary shelters or in slums, households living in *kaccha* (“temporary”) accommodation, and households having members who are transgender or who have HIV/AIDS—were also eligible for food. Households with income above the poverty line (but who did not meet the exclusion criteria) were classified as above poverty line (APL) households and were eligible for a smaller quota of food grain than the Antyodaya Anna Yojana or BPL households.

In recent years, a push to use the PDS as a vehicle to deliver fortified wheat has been made despite the lack of rigorous evaluations of whether such an intervention in the past 15 years has had any impact on anaemia among beneficiaries. Therefore, in the current impact assessment, we examined whether PDS fortified wheat distributed in two Indian states—Punjab (PN) and Tamil Nadu (TN)—between 2008 and 2012 affected Hb levels and anaemia prevalence among pregnant women, using pregnant women in neighbouring states where PDS fortified wheat was not rolled out as controls.

## 2 METHODS

### 2.1 Data sources

Data from seven national surveys were drawn upon to conduct our analyses. The Indian District Level Health Surveys (DLHS; International Institute for Population Sciences, 2006 and 2014), the largest state and district representative data on women's anaemia in India, in (a) 2002–2004 (DLHS2; International Institute for Population Sciences, 2005) and (b) 2012–13 (DLHS4; International Institute for Population Sciences, 2015), were used for Hb, covariates, and general characteristics of the pregnant women included in our analyses. These surveys are based on a representative sample of households across states and union territories in India. Within households, the target sample was all women aged 15–45 years in 2002–2004 and 15–49 years in 2012–2013. Survey respondents were selected through a stratified multistage sampling procedure conducted separately by states, districts, and urban and rural areas within districts. Further sampling methodology details are available through the survey reports (Ministry of Health and Family Welfare, 2005, 2014). The Indian National Family Health Surveys (NFHS) from (c) 1998 to 1999 (NFHS2; International Institute for Population Sciences, 2000) and (d) 2005 to 2006 (NFHS3; International Institute for Population Sciences, 2007) were used to look at trends in anaemia prior to the PDS intervention. The National Sample Surveys of Consumer Expenditure in (e) 2004–2005 (NSS‐CES61; National Sample Survey Organization, 2006); (f) 2009–2010 (NSS‐CES66; National Sample Survey Organization, 2011); and (g) 2011–2012 (NSS‐CES68; National Sample Survey Organization, 2013) were used to examine trends in the uptake of PDS commodities.

### 2.2 Study population and sample selection process

Within the DLHS2 survey, Hb levels were drawn from the “pregnant women” data set, and other characteristics and covariates were taken from the “women” data set. Women with missing or zero values for Hb, who were nonpregnant, or had missing pregnancy status were excluded before merging datasets. In DLHS2, 4,684 of 7,819 women (60%) agreed to Hb measurements, and in DLHS4, 7,218 of 7,514 women (96%) agreed. In DLHS2, those who agreed were slightly younger and less educated than those who refused (Figure S1). Only data from women in the states of interest—PN, Haryana (HR), TN, Andhra Pradesh (AP), Karnataka (KA), and Kerala (KR)—were retained. Himachal Pradesh, although neighbouring PN, was not included due to highly disparate geographical characteristics. A similar process was used for the DLHS4 survey, with Hb being drawn from the “clinical anthropometric and biochemical” data file and other characteristics from the “women” data file. DLHS4 and DLHS2 data sets were then appended to yield a final sample size of 10,186 pregnant women to be used in our analyses. This sample is a repeated cross section at the individual level and a panel at the district level.

### 2.3 Outcomes and covariates

Anaemia prevalence (Hb < 11 g/dL), defined according to World Health Organization (WHO) standards (WHO, 2011a), and mean Hb were the two outcomes of interest. Laboratory measurement of Hb was done using the dried blood spot (DBS) method (Ministry of Health and Family Welfare, 2005, 2014). Covariates included the respondent women's education (number of years of schooling); number of IFA tablets consumed; woman's age; dummy variables for households that cook with wood (Page, Patel, & Hibberd, 2015); women who were married before 18 years of age; urban households; religion of household (Hindu or Muslim); caste category of the household (scheduled caste, scheduled tribe, or other backward classes); and household wealth quintiles. Wealth quintiles were created from a principal component analysis of household socio‐economic status including type of house (impermanent, semipermanent, and permanent); drinking water source (tap inside house, shared tap, hand pump, and well); toilet facility (flush toilet, pit latrine, shared toilet, public toilet, and no toilet); lighting source (electricity and kerosene); and physical assets (radio, sewing machine, TV, phone, bicycle, motorcycle, and car; Filmer & Pritchett, 2001). We also controlled for the proportion of sampled individuals who practice open defection in a village to capture negative disease environment externalities that may influence anaemia at an ecological level (Spears, Ghosh, & Cumming, 2013).

### 2.4 Intervention description

Two Indian states, TN (in southern India) and PN (in northern India), undertook wheat flour fortification initiatives between 2008 and 2012 to address anaemia. In TN, wheat flour (locally called “atta”) was fortified and sold through public distribution fair price shops starting in 2008 and ending in 2012 (Ramakrishnan, 2012; Vydhianathan & Radhakrishnan, 2016). Subsidized wheat flour in TN was fortified with a premix consisting of vitamin A (3,300 IU/kg), folic acid (1.5 mg/kg), and iron (60 mg/kg; personal communication with Tamil Nadu Roller Flour Millers Association). Flour was fortified by private millers supplied with wheat and premixes by the government and was sold at INR 11 per kg (0.30 USD per kg at 2008 exchange rate; USD 1 = INR 40) to all entitlement‐card holding beneficiaries of the PDS in the state. The intervention was similar in PN, where fortification of wheat flour was introduced in early 2008 (Government of Punjab, 2008) and ended in mid‐2011. The premix in PN consisted of iron (30 mg/kg) and folic acid (1.5 mg/kg; Corporation, 2011; Fiedler, Babu, Smitz, Lividini, & Bermudez, 2012). Fortified wheat flour was sold at INR 12 per kg to only APL card holding beneficiaries with a quota of 35 kg per family per month (Indian Flour Fortification Network & World Food Program, 2011). Both states implemented their own variants of the Gujarat fortification model (Fiedler et al., 2012).

### 2.5 Identification strategy

Our objective was to identify the impact of introducing fortified wheat flour into the PDS on Hb and anaemia in pregnant women. Ideally, random assignment of household access to subsidized fortified wheat flour would allow causal estimation of the average intervention effect (Jensen, 2008). However, in the absence of a randomized controlled trial, the second best approach is the quasi (or natural) experiment method, which mimics a randomized allocation setting under reasonable conditions. In the current effort, we exploited the natural experiment setting where PN and TN introduced fortified wheat flour for sale through their respective PDSs.

Two methodological issues were addressed. First, because these “treatment states” self‐selected into delivering the intervention, there may be unobservable factors that drive programme placement such as a well‐functioning delivery system or willingness to innovate. Such factors, however, are likely to be fixed over the short‐term. Second, there may be time‐varying factors that could bias estimates such as food price rise in open markets or national implementation of development programmes. A common method of controlling for both issues is to use panel data and estimate difference‐in‐differences (DID) models (Galiani, Gertler, & Schargrodsky, 2005).

The DID requires selecting an appropriate control group for the treatment states, that is, states where iron‐fortified wheat was distributed through the PDS (PN and TN), and outcome data for preintervention and postintervention periods. The changes in the control group provide an estimate of the true counterfactual, that is, what would have happened to the treatment group if there had been no intervention. In our sample, neighbouring states with similar diets, demographic composition, and geographic and socio‐economic conditions served as controls for the treatment states. Thus, the change in outcomes in the treatment states between 2004 and 2013 controls for temporally fixed characteristics at the state level, and the change in outcomes in the control areas controls for time‐varying factors that are common to both control and treatment areas. The DID approach accounted for observed and unobserved time‐invariant state and district‐level characteristics that may have been correlated with the decision to introduce fortified wheat into the PDS, as well as with wheat consumption levels.

### 2.6 Parallel trends

The main identifying assumption underlying DID validity is that the change in Hb levels or anaemia in control states is an unbiased estimate of the true counterfactual. Given that this assumption is not directly testable, we tested whether preintervention secular time trends in the control and treatment states were similar. A set of DID estimations using NFHS2 and NFH3 data allowed us to empirically test whether Hb levels and anaemia moved in tandem in the preintervention periods across the treatment and control states.

### 2.7 Estimating equations

To determine the causal effect of introducing fortified wheat in the PDS, we estimated Equation 1.

(1) $$Y=\beta_{0}+\beta_{1}\text{Trend}+\beta_{2}\text{Intervention}+\beta_{3}\mathrm{DID}+X+Ɛ$$

In Equation 1, *Y* is the outcome of interest (Hb level or anaemia prevalence) in each state in each time period. “Trend” is a time dummy that takes value 0 for data points obtain from DLHS2 and 1 for DLHS4; “Intervention” is a treatment state dummy that takes value 0 for control states (HR, AP, KR, and KA) and 1 for treatment states (PN and TN); and DID is the interaction of the time and treatment dummies. β_0_ is the baseline average, β_1_ represents the time trend in the control group, β_2_ represents the differences between the treatment and control states at baseline, and β_3_ represents the difference in changes over time or the average treatment effect. β_3_, the DID estimator, is the coefficient of interest and, if statistically significant (*P* < 0.05), then the null hypothesis that the introduction of PDS wheat had no impact on Hb levels or anaemia prevalence was rejected. *X* is the set of household and women specific covariates, and Ɛ is a white‐noise term that represents residual variation. Standard errors were clustered at the district level to control for intradistrict correlations. Clustering was done at the district level due to implementation and programme exposure variation at this level.

To empirically test for parallel trends, Equation 1 was modified by excluding *X* and replacing the dummy “Trend” with a variable that takes the value 1 for NFHS2 and 7 for NFHS3, given that these data sets are separate by 7 years. In this modified model, if the coefficient β_3_ is not statistically significant (*P* > 0.05), then the null hypothesis of parallel trends in treatment and control states was not rejected.

### 2.8 Robustness checks

There are three other concerns that may lead to biased DID estimates. First, due to imperfect targeting in the PDS, there may be differential impacts across socio‐economic groups. APL households were directly targeted by the fortification intervention in PN, but in the context of imperfect targeting, it is likely that poor (non‐APL) households would have purchased some PDS wheat. Other work has also shown that iron status is generally lower in poorer groups (Balarajan, Fawzi, & Subramanian, 2013) and, due to the inverse relation between iron status and iron absorption (Hallberg & Hulthén, 2000), the intervention impact may differ across income classes. Due to unavailability of APL entitlement card status in the DLHS data, we instead stratified the sample by wealth quintile, comparing the effect of the intervention on the top three quintiles (nonpoor) to the effect of the intervention on the bottom two quintiles (poor) using a triple difference model for PN (Equation 2).

(2) $$Y=\alpha_{0}+\alpha_{1}\text{Trend}+\alpha_{2}\text{Intervention}+{\alpha_{3}}^{“}\text{non}-{\text{poor}}^{”}+\alpha_{4}\mathrm{DID}+\alpha_{5}\text{Trend}\cdot {}^{“}\text{non}-{\text{poor}}^{”}+{\alpha_{6}}^{“}\text{non}-{\text{poor}}^{”}\cdot \text{Intervention}+\alpha_{7}\mathrm{DDD}+X+Ɛ$$

Equation 2 is like Equation 1 but has four additional coefficients, where α_7_ represents the differential impact of the treatment on nonpoor households in treatment states.

Second, although neighbouring states may be generally similar to treatment states, heterogeneity may still exist due to large geographical area. Thus, we estimated models using Equation 1, but instead of using neighbouring states, we used bordering districts as control groups. Restricting the control states in this manner forces control and treatment groups to be geographically proximal. However, using bordering districts carries with it the inherent concern of potential spillover effects. Spillover effects may arise if high levels of leakage or pilferage of PDS wheat flour in treatment states are present. Literature on state‐specific leakage rates shows large amounts of PDS cereal grain leakage in PN (60% of the allocated grains did not reach households) but not in TN (10%; Dreze & Khera, 2015b). Therefore, as a third robustness check, we ran a set of models using nonbordering districts from neighbouring states.

## 3 RESULTS

### 3.1 Intervention coverage

Prior to the intervention, which started in 2008, virtually no households in PN and only 11% of households in TN consumed any PDS wheat (Table 1). One year after the intervention began, coverage increased to 15% in PN and 54% in TN, and the level of coverage held by 2011, 1 year prior to the end of the intervention. In PN, total wheat consumption was relatively high and remained stable before and after the introduction of PDS wheat. Furthermore, in PN, PDS wheat as a proportion of total wheat consumed increased from 0% before the intervention to 10% after the intervention. Additionally, a very small fraction (1–3%) of APL cardholders in PN made any purchases of PDS wheat, suggesting a lack of demand. The substantial increase in coverage in TN was accompanied by an increase in both the total wheat consumed and the proportion of the total that was PDS wheat so that, by 2011, total wheat consumption doubled and the share of PDS wheat consumed increased fivefold. This translates to approximately 29,000 tons of wheat consumed per month at the population level in TN. In addition, almost one third of the households in TN across all wealth quintiles were APL cardholders that purchased PDS wheat, compared with 1–3% in PN.

**Table 1:** Coverage and consumption of public distribution system cereals in treatment states^a^

	Punjab			Tamil Nadu		
	2004	2009	2011	2004	2009	2011
Coverage						
Consumed any PDS rice, %	0	0	0	63	78	78
Consumed any PDS wheat, %	0	15	16	11	54	56
Quantity consumed						
Total rice consumed per‐capita per month, kg	0.94	0.99	1.07	9.56	9.18	8.40
Total wheat consumed per‐capita per month, kg	8.60	8.01	7.74	0.36	0.58	0.65
Share of PDS						
Share of PDS rice out of total rice consumed, %	0	2	2	34	45	45
Share of PDS wheat out of total wheat consumed, %	0	10	10	11	51	53
Share of population with APL cards purchasing wheat from PDS, %^b^						
Expenditure quintile 1 (Q1)	0	—	3	7	—	29
Q2	0	—	1	9	—	39
Q3	0	—	2	9	—	38
Q4	0	—	1	10	—	37
Q5	0	—	1	9	—	30
Observations	4,288	3,115	3,118	8,296	6,638	6,647

*Note*. APL: above poverty line; PDS: public distribution system.

a Data source: NSSO‐CES rounds 61 (2004), 66 (2009), and 68 (2011).

b Wealth quintiles were defined using monthly per capita consumption expenditure.

### 3.2 Sample characteristics at baseline

Haemoglobin did not differ between PN and HR, with rates of anaemia among pregnant women over 90% in both states (Table 2). Respondents were 23 years of age on average. In HR, compared with PN, more households cooked with wood, early marriage was more common, more household heads were Hindu, fewer household heads were schedule tribe, and more household heads were other backward caste. Wealth was similar between states. After restricting the sample to bordering districts, Hb among the sample from HR was 1 g/dL higher, and anaemia prevalence was 7% lower than in PN. No significant differences in the primary outcomes were evident when looking at nonbordering districts. Patterns among covariates in the bordering and nonbordering comparisons generally mimicked the statewide comparison.

**Table 2:** Summary of baseline (2004) levels of key outcomes and covariates, Punjab versus Haryana

Comparison	1. Statewide		2. Bordering districts		3. Nonbordering districts	
	Treatment (PN)	Control (HR)	Treatment (PN)	Control (HR)	Treatment (PN)	Control (HR)
Pregnant women, No.	702	885	175	251	527	634
Hb, g/dL	9.4 (5.5)	8.9 (5.3)	7.8 (1.8)	8.7 (3.2)***	9.9 (6.3)	9.0 (5.9)
Anaemia prevalence, %^a^	90	92	99	92*	87	92
Years of schooling, No.	6.4 (5.2)	6.0 (5.0)	5.3 (5.2)	5.9 (4.8)	6.8 (5.1)	6.1 (5.1)
IFA tablets consumed, No.	20 (43)	20 (39)	16 (43)	22 (45)	22 (42)	19 (37)
Age, years	23.3 (4.1)	23.0 (4.1)	22.9 (4.3)	23.0 (3.9)	23.5 (4.07)	23.0 (4.2)
HH cooks with wood, %	42	57*	47	49	41	61*
Married <18 years, %	18	38***	23	29	17	42***
HH is urban, %	28	25	29	22	28	26
HH head is Hindu, %	36	86***	27	84***	39	86***
HH head is Muslim, %	1.8	8.4	0.0	1.6	2.5	11.0
HH head is SC, %	45	27***	47	33**	44	25***
HH head is ST, %	1.4	0.7	0.6	1.6	1.7	0.3
HH head is OBC, %	17	35***	14	34**	18	35**
Wealth quintile	2.17 (1.31)	2.24 (1.22)	2.01 (1.27)	2.32 (1.18)	2.23 (1.32)	2.21 (1.24)

*Note*. IFA: iron folic acid; Hb: haemoglobin; HH: household; HR: Haryana; OBC: other backward classes; PN: Punjab; SC: scheduled caste; ST: scheduled tribe. Data are presented as mean (SD) or percentages.

a Anaemia defined as haemoglobin <11 g/dl per World Health Organization criteria for pregnant women [3].

* *P* < 0.05.

** *P* < 0.01.

*** *P* < 0.001 compared with treatment.

Hb and anaemia among pregnant women in TN versus its neighbouring states—AP, KR, and KA—did not differ (Table 3). However, the treatment and control states differed in all other dimensions at baseline, with women in the control arm having significantly lower education, consuming more IFA tablets, marrying earlier, living in less urban areas, and cooking with wood more frequently. Differences in religious and caste composition were also found, with fewer Hindu and more Muslim household heads, as well as fewer scheduled caste and other backward caste but more scheduled tribe household heads in the control arm. The differences were subtler or were lost when comparing bordering districts and were similar to those in the statewide comparison when comparing nonbordering districts, as expected.

**Table 3:** Summary of baseline (2004) levels of key outcomes and covariates, Tamil Nadu versus Andhra Pradesh, Kerala, and Karnataka

Comparison	1. Statewide		2. Bordering districts		3. Nonbordering districts	
	Treatment (TN)	Control (AP, KR, KA)	Treatment (TN)	Control (AP, KR, KA)	Treatment (TN)	Control (AP, KR, KA)
Pregnant women, No.	1283	1806	377	334	906	867
Hb, g/dL	10.4 (6.1)	10.0 (4.9)	12.0 (7.9)	10.8 (4.9)	9.7 (5.0)	10.0 (4.8)
Anaemia prevalence, %^a^	86	87	75	81	90	87
Years of schooling, No.	7.1 (4.7)	5.8 (4.9)***	8.0 (4.6)	7.7 (4.5)	6.7 (4.7)	5.9 (4.9)
IFA tablets consumed, No.	23 (42)	37 (65)***	25 (45)	33 (61)	23 (40)	33 (60)**
Age, years	23.4 (4.0)	22.4 (4.5)***	23.7 (3.8)	23.4 (4.9)	23.2 (4.0)	22.7 (4.7)
HH cooks with wood, %	65	73*	57	73*	68	75
Married <18 years, %	21	49***	16	31*	24	52***
HH is urban, %	38	29***	46	28***	35	30
HH head is Hindu, %	90	77***	87	69**	92	77***
HH head is Muslim, %	6	18***	5	20*	6	19***
HH head is SC, %	29	20***	23	20	32	19***
HH head is ST, %	2.0	6.6***	1.3	5.1	2.2	8.9***
HH head is OBC, %	68	46***	75	49***	65	44***
Wealth quintile	2.45 (1.40)	2.27 (1.31)*	2.66 (1.38)	2.07 (1.21) ^***^	2.37 (1.39)	2.28 (1.31)

*Note*. AP: Andhra Pradesh; IFA: iron folic acid; Hb: haemoglobin; HH: household; KA: Karnataka; KR: Kerala; OBC: other backward class; SC: scheduled caste; ST: scheduled tribe. Data are presented as mean (SD) or percentages.

a Anaemia defined as haemoglobin <11 g/dl per World Health Organization criteria for pregnant women [3].

* *P* < 0.05.

** *P* < 0.01.

*** *P* < 0.001 compared with treatment.

### 3.3 Parallel trends in anaemia prior to the intervention

**Figure 1:**
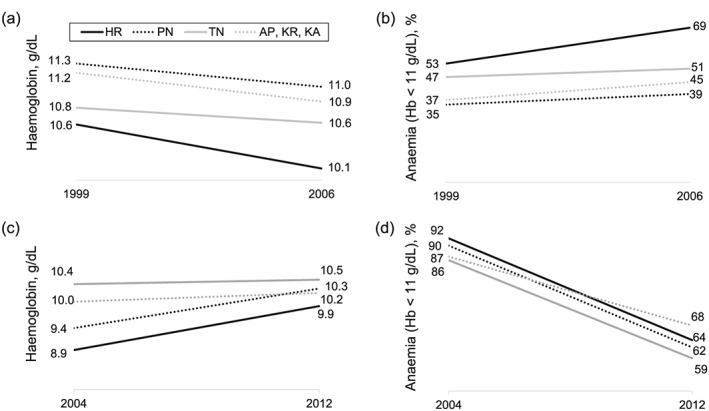
Statewide trends in haemoglobin level and anaemia prevalence among pregnant women in India. Two comparisons were made between: (1) Punjab (treatment; solid black lines) versus Haryana (control; dotted black lines) and (2) Tamil Nadu (treatment; solid grey lines) versus Andhra Pradesh, Kerala, and Karnataka combined (control; dotted grey lines). Panels a and b show Hb and anaemia trends prior to the intervention using data from the National Family Health Surveys (NFHS). Panels c and d show trends preintervention and postintervention using data from District Level Household Surveys (DLHS), which were the data used in our econometric models to assess impact. Note that different haemoglobin measurement methods are used in NFHS and DLHS; NFHS uses Hemocue, whereas DLHS uses the dried blood spot, hence the different Hb and anaemia magnitudes in panels a and b versus in panels c and d

Parametric tests confirmed that trends in Hb and anaemia were parallel for the treatment and control states prior to the wheat fortification effort (Figure 1a,b; Table S1). In all states, Hb declined and anaemia increased over the 7‐year period between NHFS2 and NFHS3, and the rate of change did not differ between treatment and control states (*P* > 0.05 in all comparisons).

### 3.4 Impact of iron‐fortified wheat distribution on haemoglobin and anaemia prevalence

#### 3.4.1 Haemoglobin and anaemia prevalence before and after iron‐fortified wheat distribution

Mean Hb increased by 0.9–1.0 g/dL in PN and HR, and by 0.1–0.2 g/dL in TN and AP, KR, and KA. Anaemia prevalence decreased by 28% in both PN and HR; by 27% in TN; and by 19% in AP, KR, and KA (Figure 1c,d). The Hb distribution of sampled pregnant women shifted right (Hb increased) in both treatment and control states from 2004‐2005 to 2011–2012 (Figure S2).

#### 3.4.2 Double‐difference estimates

The analysis for PN versus HR yielded no significant DID coefficients across all model specifications (Tables 4 and S2). DID regressions for TN versus AP, KR, and KA yielded significant beta coefficients for the interaction term (Tables 4 and S3) in the anaemia models; that is, whereas anaemia decreased significantly in both groups, the decrease was greater by 8% in TN compared with that in AP, KR, and KA. However, this finding was lost in the comparison of bordering districts, as would be expected if there were spillover effects. Factors associated with lower mean Hb and higher anaemia prevalence included early marriage and being in the scheduled caste group, whereas having more years of education, being older, and living in an urban rather than a rural setting were protective factors.

**Table 4:** Impact of fortifying wheat through the PDS on haemoglobin level and anaemia prevalence in pregnant Indian women

Comparison	Outcome	Time	Treatment	Treatment, time interaction	Significant covariates^a^
		β_1_ (SE)	β_2_ (SE)	β_3_ (SE)	Covariate (β)
Statewide					
PN vs. HR	Hb	1.00 (0.4)**	0.44 (0.6)	−0.18 (0.7)	Married <18 years (−0.39), SC (−0.79)
	Anaemia^b^	−0.25 (0.0)***	−0.01 (0.0)	−0.01 (0.1)	Education years (−0.01**), wealth Q3 (0.06*), SC (0.12**)
TN vs. AP, KR, KA	Hb	−0.02 (0.3)	0.050 (0.5)	−0.00 (0.5)	Education yrs (0.03*), age (0.04**), wealth Q2 (0.52*)
	Anaemia^b^	−0.17 (0.0)***	−0.02 (0.0)	−0.08 (0.0)*	Age (−0.004***)
Bordering districts					
PN vs. HR	Hb	1.48 (0.5)*	−0.62 (0.2)*	0.64 (0.6)	Education yrs (0.08*)
	Anaemia^b^	−0.36 (0.1)***	−0.03 (0.0)	0.08 (0.1)	None
TN vs. AP, KR, KA	Hb	0.20 (0.9)	1.63 (1.1)	−1.30 (1.4)	Age (0.08**), SC (−1.15**)
	Anaemia^b^	−0.20 (0.1)*	−0.10 (0.1)	0.01 (0.1)	No. IFA tablets (0.00**)
Nonbordering districts					
PN vs. HR	Hb	−0.78 (0.4)	0.70 (0.8)	−0.46 (0.9)	Married <18 yrs (−0.53*), SC (−0.99*)
	Anaemia^b^	−0.22 (0.0)***	−0.02 (0.1)	−0.03 (0.1)	Wealth Q3 (0.07*), wealth Q4 (0.06*), SC (0.13*)
TN vs. AP, KR, KA	Hb	−0.06 (0.3)	−0.06 (0.5)	0.50 (0.4)	Wealth Q2 (0.56**)
	Anaemia^b^	−0.17 (0.0)***	0.02 (0.0)	−0.09 (0.0)*	Age (−0.003*), urban (−0.04*), SC (−0.04*)

*Note*. AP: Andhra Pradesh; IFA: iron folic acid; Hb: haemoglobin; HH: household; HR: Haryana; KA: Karnataka; KR: Kerala; PN: Punjab; Q: quintile; SC: scheduled caste.

a A complete table of beta coefficients and standard errors for nonsignificant covariates can be found in the the Supporting Information.

b Anaemia defined as haemoglobin <11 g/dl per World Health Organization criteria for pregnant women [3].

* *P* < 0.05.

** *P* < 0.01.

*** *P* < 0.001.

#### 3.4.3 Triple‐difference estimates by wealth quintiles

No significant differential impacts of wheat fortification by wealth status were found in the comparison of PN versus HR (Table S4) in the triple difference model.

## 4 DISCUSSION

### 4.1 Interpretation of current findings

The mean Hb count of pregnant women living in PN and HR increased significantly from 2002–2004 to 2012–2013, resulting in a decline in anaemia prevalence during this 11‐year period. Results from our DID model, however, revealed that the provision of fortified wheat flour through PDS in PN did not lead to a significant additional improvement in average Hb or any incremental decline in the incidence of anaemia in the state. This result is robust to comparisons between the entire states of PN and HR, only bordering districts of the two states and only nonbordering districts.

Fortified wheat was targeted to the APL households in PN, allowing us to estimate a triple difference model where we compare APL and BPL households of PN. These results also showed no significant treatment effect on Hb or anaemia in the targeted subpopulation.

Unlike PN, fortified wheat flour introduction in the PDS resulted in an additional 8% decline in anaemia in TN compared with its neighbouring states. The effect disappeared when the sample was restricted to only bordering districts, which may reflect spillover of fortified flour from TN to bordering areas. Our comparison of nonbordering districts supports this spillover hypothesis; we found that the intervention effect was marginally larger (9% vs. 8%) in nonbordering districts compared with the statewide comparison.

Despite finding an impact on anaemia, we did not find a significant impact of the intervention on mean Hb in pregnant women in TN. One possibility is that fortified flour consumed by women whose Hb was just below the WHO threshold led to an increase in their Hb, which brought them over the threshold. If so, even small Hb improvements in a fraction of the total population would be visible in the anaemia data. Without a dataset containing information on both Hb and household consumption patterns, we cannot ascertain if this was indeed the mechanism at work. Low effectiveness of iron‐fortified wheat flour in terms of improving Hb may also reflect a large proportion of anaemia being due to causes other than iron deficiency such as inherited genetic disorders or other nutritional deficiencies.

In our study, the provision of fortified wheat flour in PDS had no significant effect on Hb and anaemia prevalence among pregnant women in APL households of PN, perhaps because only 1% of APL households in the state purchase wheat (or wheat flour) from PDS shops (Table 1). Wheat is the staple food in PN, with per capita daily consumption around 250 g/day, but almost all of it is purchased from open markets or sourced from own production, especially by the APL households. Given the high consumption of wheat in PN, if fortified wheat was actually consumed, one would expect a significant improvement in iron status based on evidence from efficacy trials (Hurrell et al., 2010). A similar intervention in TN also had only a small effect, perhaps because of low consumption of wheat flour in the state. Evidence from a fortification programme in Costa Rica where per capita wheat flour consumption is around 74 g/day provides a useful comparison. In this programme, wheat flour, maize flour, and milk were all fortified with iron. An impact assessment showed an 8% reduction in anaemia prevalence among women of reproductive age from preprogramme levels (Martorell et al., 2015). However, with a mean wheat flour consumption level of just 21 g/day in TN, one would expect a very small impact of the fortification on iron status, if any. In comparison, per capita rice consumption in TN, at 280 g/day, is 13 times higher than per capita wheat consumption. Thus, the fortification programme may have used the wrong distribution channel in PN and targeted the wrong food item in TN.

### 4.2 Study strengths

Our study had three main strengths: the quasi‐experimental design using sound econometric methods, accounting for potential spillovers, and the use of a population with a high anaemia burden.

The PDS is India's largest welfare programme and is designed to guarantee that two thirds of India's population receives food as a fundamental right. Given its scale, the use of the PDS platform to deliver health‐ and nutrition‐related interventions has large potential for impact, making research on the effectiveness of such interventions crucial. However, the scale of the programme as well as political and ethical considerations make it infeasible to design and conduct randomized trials requiring a control group. In the absence of experimental data on cereal fortification programmes in India, we used pre‐ and post‐cross‐sectional surveys with identifiable treatment and comparison groups to estimate fortification's impact on Hb and anaemia. Because both rounds of DLHS use the same sampling strategy, we could apply panel data techniques to repeated cross‐sectional data. Parallel trends between treatment and control groups in Hb levels and anaemia prevalence in the preintervention period created a natural experiment that we exploited to estimate the effect of flour fortification in the treatment states, PN and TN. To the best of our knowledge, this is the first such attempt to assess the impact of a large fortification programme in India using econometric methods.

Possible spillovers of fortified flour to households in control groups—households in bordering districts of nontarget states, or the nontargeted BPL households—could potentially lead to a downward bias in the estimated fortification effect. The DLHS uses a large sample that is representative at the district level, which allowed us to address the spillover concern by comparing nonadjacent districts of treatment and control states where chances of spillover are low. That we found similar results when comparing the entire states or only nonbordering districts gives us confidence in our main finding of low or no effect of flour fortification on anaemia.

### 4.3 Study limitations

The main limitation of this paper is the potential for confounding effects due to the gap between surveys. The DID analysis covered a long period (nearly 10‐year gap between preobservation and postobservation), which render the estimates susceptible to time‐varying confounders. For instance, there were improvements in the functioning of the PDS, public health system, and various social assistance programmes such as pension schemes during the study period, with variation across states in the extent of these improvements. Correlation between such state‐level changes and introduction of flour fortification would yield biased estimates of the effect of fortification. To mitigate these concerns, in PN, we used a narrower definition of treatment group (only targeted APL households) and use triple‐difference design. In TN, however, the small effect of fortification could be due to expansion and improvements in the delivery of public health and social welfare schemes in the state during the periods between DLHS2 and DLHS4. We could not use the triple‐difference method in TN because the programme was not targeted to any subgroup.

### 4.4 Lessons learned and recommendations for policymakers

Recently, other states of India such as Madhya Pradesh and Rajasthan have launched initiatives to fortify food items such as wheat flour and edible oil. From our research on flour fortification in PN and TN, we can draw several important lessons, as food fortification programmes in India and other countries are designed and implemented at scale.

First, fortification programmes should choose appropriate commodities to fortify based on an in depth understanding of local dietary habits. The choice should not be guided purely by technological and logistical considerations. Fortified rice may have had a larger effect in a predominantly rice‐eating state such as TN. Rice consumption is higher than wheat consumption in 24 out of 35 states of India. Some rice is consumed in all states, whereas per capita wheat consumption is less than 20 g/month in 12 states. Given the importance of rice in Indian diets, the government may want to explore more cost‐effective ways to fortify rice.

Second, marketing of fortified food should be informed by data on local purchasing practices and consumer behaviour. In the PDS, a popular channel for distributing fortified food in India, coverage varies widely from state to state, commodity to commodity, between rural and urban, and poor and nonpoor households. Nationally, 44% of households reported buying any rice or wheat from PDS in 2011–2012, compared with 16% in PN. Furthermore, 30% of total rice purchased in India comes from PDS compared with only 17% of wheat. Thus, PDS is not the right channel to distribute all fortified foods. Additionally, most consumers bring their own wheat to the flourmill in PN, but, in TN, they buy premilled flour instead. Therefore, in areas where consumers bring their own wheat for milling, we recommend government provision of a ready fortificant premix to local mills.

Third, there are real challenges in both the implementation and the documentation of government schemes. Governments in India have a poor record in implementing targeted schemes (Dreze & Khera, 2015a; Niehaus, Atanassova, Bertrand, & Mullainathan, 2013), and anaemia is prevalent across all income groups (Balarajan et al., 2013). We advocate, therefore, that fortification programmes be universal in scope. Also, state governments seldom monitor food‐based interventions, and information pertaining to factors such as quality control and uptake is scarce. We suggest that routinized monitoring be embedded as a standard practice to track supply chains from mills to ration shops.

Fourth, the provision of fortified food items needs to be supported with campaigns to raise awareness about their potential benefits and increase demand. Fortified wheat flour is indistinguishable in taste, colour, and odour from unfortified flour (Muthayya et al., 2012). Thus, the low consumer demand for this product in both PN and TN as well as other states (Bhagwat, Gulati, Sachdeva, & Sankar, 2014) despite subsidies is not likely due to tangible differences. A more plausible explanation is low awareness about anaemia and the fortified flour as a remedy to this public health epidemic.

Given that food subsidy programmes are popular globally, the potential for impacting anaemia through these platforms is high. Governments worldwide would benefit from the policy recommendations outlined here.

## 5 CONCLUSIONS

Anaemia remains a major public health issue despite decades of concerted effort to reduce its burden. More than 75 countries fortify flour, but the evidence of its effectiveness in reducing the prevalence of anaemia is limited, particularly in India (Pachón, Spohrer, Mei, & Serdula, 2015). Governments intending to use fortification as a strategy to combat anaemia should keep in mind that programmes are likely to be more effective if the food being fortified is regularly consumed in adequate quantities by the target population and is distributed through popular outlets. Failure to account for these factors may underlie our finding of no impact of wheat fortification on Hb in pregnant Indian women.

## CONFLICTS OF INTEREST

The authors declare that they have no conflicts of interests.

## CONTRIBUTIONS

All authors contributed equally to this manuscript.

## Supporting information


**Figure S1.** **District Level Household Survey strategy for hemoglobin measurement among pregnant Indian women, with age and education comparison by consent status.** Relatively more women refused in DLHS2 (2004) compared to DLHS4 (2012), with those refusing being slightly older and more educated than those agreeing to have their hemoglobin measured.
**Figure S2. Kernel density plots of hemoglobin in treatment and control states prior to and after the iron‐fortified wheat distribution**. Solid lines represent 2004 data and dotted lines represent 2011 data. A vertical reference line at 11 g/dL indicates the WHO cutoff for anemia in pregnant women. Comparisons were made between: 1) Punjab (treatment; panel A) vs. Haryana (control; panel B) and 2) Tamil Nadu (treatment; panel C) vs. Andhra Pradesh, Kerala, and Karnataka combined (control; panel D).
**Table S2. Difference‐in‐difference estimate of fortifying wheat through the PDS on anemia ‐ Punjab vs Haryana**
**Table S3. Difference‐in‐difference estimate of fortifying wheat through the PDS on anemia ‐ Tamil Nadu vs Andhra Pradesh, Kerala and Karnataka**
**Table S4. Triple‐difference estimates ‐ Punjab versus Haryana**
