# Supplementary material for: Impact of subsidized fortified wheat on anaemia in pregnant Indian women
Source: Matern Child Nutr. 2018 Sep 4;15(1):e12669. doi: 10.1111/mcn.12669 (PMC6585702; doi:10.1111/mcn.12669)
Supplement: Supplementary file 1 — Figure S1. District Level Household Survey strategy for hemoglobin measurement among pregnant Indian women, with age and education comparison by consent status. Relatively more women refused in DLHS2 (2004) compared to DLHS4 (2012), with those refusing being slightly older and more educated than those agreeing to have their hemoglobin measured. Figure S2. Kernel density plots of hemoglobin in treatment and control states prior to and after the iron‐fortified wheat distribution. Solid lines represent 2004 data and dotted lines represent 2011 data. A vertical reference line at 11 g/dL indicates the WHO cutoff for anemia in pregnant women. Comparisons were made between: 1) Punjab (treatment; panel A) vs. Haryana (control; panel B) and 2) Tamil Nadu (treatment; panel C) vs. Andhra Pradesh, Kerala, and Karnataka combined (control; panel D). Table S2. Difference‐in‐difference estimate of fortifying wheat through the PDS on anemia ‐ Punjab vs Haryana Table S3. Difference‐in‐difference estimate of fortifying wheat through the PDS on anemia ‐ Tamil Nadu vs Andhra Pradesh, Kerala and Karnataka Table S4. Triple‐difference estimates ‐ Punjab versus Haryana [file MCN-15-e12669-s001.docx]

| **Supplementary Table 1. Parametric test for parallel trends^a^** | | |
| --- | --- | --- |
|  | **PN & neighboring states** | **TN & neighboring states** |
| **Model 🡪** | **(1)** | **(2)** |
| Outcome: haemoglobin (g/dL) | | |
| Parallel trends (=treatment dummy*dummy for 2005-06) | 0.039 | 0.026 |
|  | (0.0) | (0.0) |
| Treatment dummy (=1 for Punjab or Tamil Nadu) | 0.646** | -0.419** |
|  | (0.2) | (0.1) |
| Dummy for 2005-06 | -0.082* | -0.054** |
|  | (0.0) | (0.0) |
| Constant | 10.708*** | 11.260*** |
|  | (0.2) | (0.1) |
| R^2^ | 0.060 | 0.015 |
| N | 621 | 1664 |
| Outcome: Anaemia (%)^b^ | | |
| Parallel trends (=treatment dummy*dummy for 2005-06) | -0.019 | -0.007 |
|  | (0.0) | (0.0) |
| Treatment dummy (=1 for Punjab or Tamil Nadu) | -0.165** | 0.109** |
|  | (0.1) | (0.0) |
| Dummy for 2005-06 | 0.027** | 0.013* |
|  | (0.0) | (0.0) |
| Constant | 0.503*** | 0.354*** |
|  | (0.0) | (0.0) |
| R^2^ | 0.070 | 0.010 |
| N | 651 | 1778 |
| ^a^Source: NFHS data corresponding to years 1998-99 and 2005-06.  ^b^Anemia defined as hemoglobin < 110 g/L per World Health Organization criteria for pregnant women [3].  Standard errors in parentheses  + p<0.10, * p<0.05, ** p<0.01, *** p<0.001  PN, Punjab; TN, Tamil Nadu | | |

| **DLHS 2 (N=7819 pregnant women)** |  | **DLHS 4 (N=7514 pregnant women)** |
| --- | --- | --- |
| \|  \| \| --- \| |  | \|  \| \| --- \| |
| N=4684 were measured |  | N=7218 were measured |
| N=272 parent refused |  | N=0 parent refused |
| N=679 self refused |  | N=40 self refused |
| N=841 not present |  | N=38 not present |
| N=1342 others |  | N=0 others |
| N=1 missing |  | N=0 missing |
| \|  \| \| --- \| |  | \|  \| \| --- \| |
| **Age in years** |  | **Age in years** |
| Measured = 22.9 |  | Measured = 28.4 |
| Parent refused = 23.6 |  | Parent refused = . |
| Self refused = 23.8 |  | Self refused = 26 |
| Not present = 23.1 |  | Not present = 27.6 |
| Others = 22.7 |  | Others = . |
| Missing = 24 |  | Missing = . |
| \|  \| \| --- \| |  | \|  \| \| --- \| |
| **Education in years** |  | **Education in years** |
| Measured = 8.9 |  | Measured = 9.5 |
| Parent refused = 9.8 |  | Parent refused = . |
| Self refused = 9.8 |  | Self refused = 10.2 |
| Not present = 9.1 |  | Not present = 9.4 |
| Others = 8.8 |  | Others = . |
| Missing = 4 |  | Missing = . |
| \|  \| \| --- \| |  | \|  \| \| --- \| |
| **N=4676 valid Hb measurements** |  | **N=6552 valid Hb measurements** |

**Supplemental Figure 1. District Level Household Survey strategy for hemoglobin measurement among pregnant Indian women, with age and education comparison by consent status.** Relatively more women refused in DLHS2 (2004) compared to DLHS4 (2012), with those refusing being slightly older and more educated than those agreeing to have their hemoglobin measured.


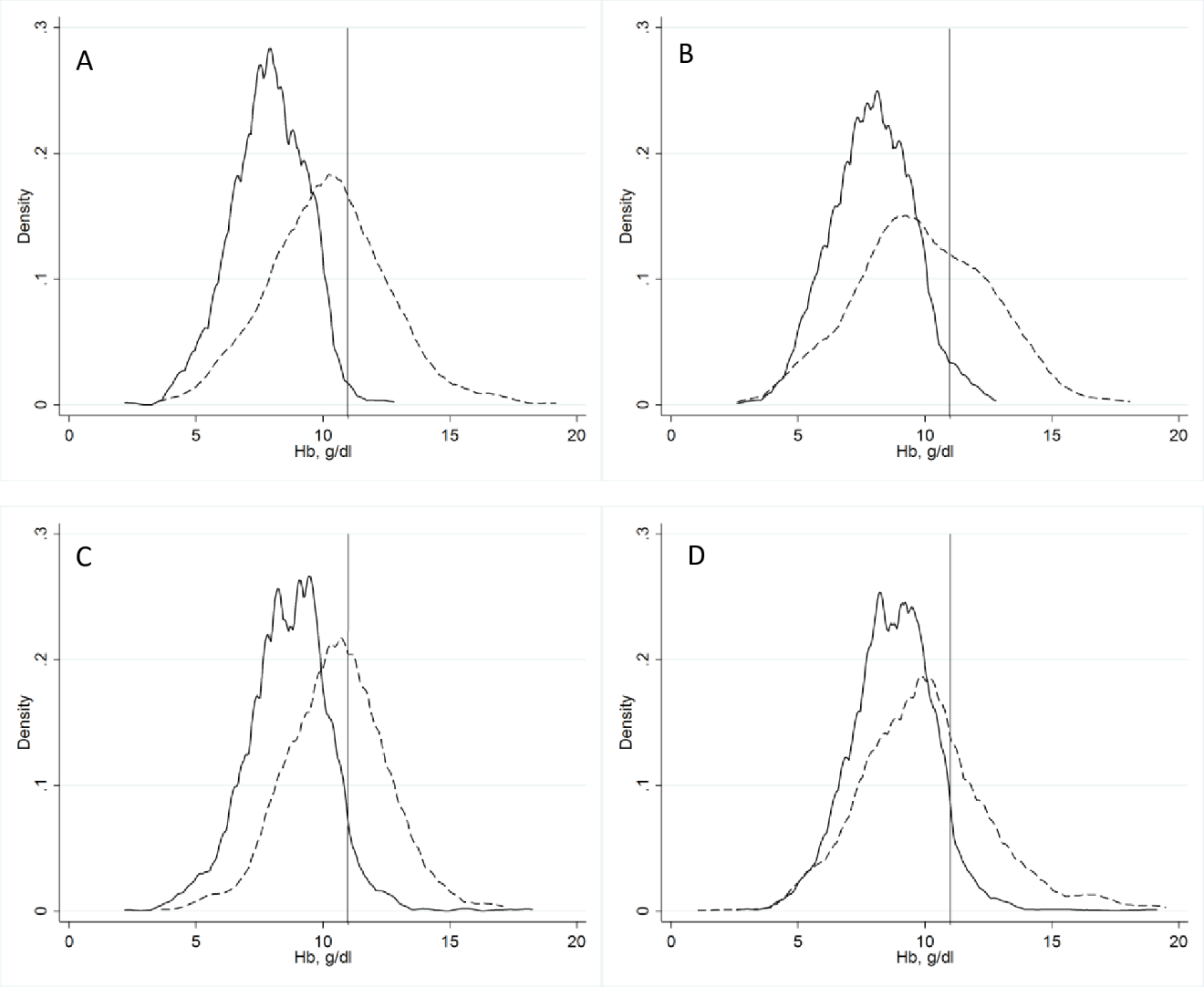


**Supplemental Figure 2. Kernel density plots of hemoglobin in treatment and control states prior to and after the iron-fortified wheat distribution**. Solid lines represent 2004 data and dotted lines represent 2011 data. A vertical reference line at 11 g/dL indicates the WHO cutoff for anemia in pregnant women. Comparisons were made between: 1) Punjab (treatment; panel A) vs. Haryana (control; panel B) and 2) Tamil Nadu (treatment; panel C) vs. Andhra Pradesh, Kerala, and Karnataka combined (control; panel D).

| **Supplementary Table 2. Difference-in-difference estimate of fortifying wheat through the PDS on anemia - Punjab vs Haryana** | | | | | | |
| --- | --- | --- | --- | --- | --- | --- |
|  | **PN vs HR** | | **PN vs HR - bordering districts** | | **PN vs HR - non-bordering districts** | |
| **Model 🡪** | **(1)** | **(2)** | **(3)** | **(4)** | **(5)** | **(6)** |
| **Predictor Outcome 🡪** | **Hb** | **Anaemia^a^** | **Hb** | **Anaemia^a^** | **Hb** | **Anaemia^a^** |
| Diff-in-diff estimator (treatment x post-treatment time dummy) | -0.184 | -0.009 | 0.643 | 0.082 | -0.457 | -0.029 |
|  | (0.7) | (0.1) | (0.6) | (0.1) | (0.9) | (0.1) |
| Treatment dummy (=1 for Punjab) | 0.438 | -0.010 | -0.621* | 0.034 | 0.698 | -0.021 |
|  | (0.6) | (0.0) | (0.2) | (0.0) | (0.8) | (0.1) |
| Post treatment year (=1 if year is 2012-13) | 1.002** | -0.252*** | 1.483* | -0.363*** | 0.780+ | -0.222*** |
|  | (0.4) | (0.0) | (0.5) | (0.1) | (0.4) | (0.0) |
| Woman's years of education | 0.036+ | -0.006** | 0.082* | -0.008+ | 0.012 | -0.005+ |
|  | (0.0) | (0.0) | (0.0) | (0.0) | (0.0) | (0.0) |
| Woman's age | 0.034+ | -0.004* | 0.035 | -0.003 | 0.028 | -0.004+ |
|  | (0.0) | (0.0) | (0.0) | (0.0) | (0.0) | (0.0) |
| Number of IFA tablets consumed by woman | 0.003 | 0.000 | 0.001 | 0.000 | 0.003 | 0.000 |
|  | (0.0) | (0.0) | (0.0) | (0.0) | (0.0) | (0.0) |
| Household cooking fuel is wood | 0.273+ | -0.010 | 0.180 | -0.027 | 0.359+ | -0.006 |
|  | (0.1) | (0.0) | (0.2) | (0.0) | (0.2) | (0.0) |
| Woman was married before 18 years of age | -0.392* | 0.009 | 0.089 | -0.033 | -0.528* | 0.022 |
|  | (0.2) | (0.0) | (0.2) | (0.0) | (0.2) | (0.0) |
| Lowest wealth quintile (base category) | 0.000 | 0.000 | 0.000 | 0.000 | 0.000 | 0.000 |
|  | (.) | (.) | (.) | (.) | (.) | (.) |
| Second lowest wealth quintile | -0.132 | 0.005 | 0.441 | -0.001 | -0.277 | -0.003 |
|  | (0.5) | (0.0) | (0.4) | (0.0) | (0.6) | (0.0) |
| Middle wealth quintile | -0.482 | 0.060* | -0.240 | 0.026 | -0.522 | 0.068* |
|  | (0.4) | (0.0) | (0.3) | (0.1) | (0.5) | (0.0) |
| Second highest wealth quintile | -0.194 | 0.039 | 0.346 | -0.016 | -0.424 | 0.056* |
|  | (0.4) | (0.0) | (0.3) | (0.1) | (0.5) | (0.0) |
| Highest wealth quintile | -0.105 | 0.022 | 0.390 | -0.068 | -0.304 | 0.049+ |
|  | (0.5) | (0.0) | (0.3) | (0.1) | (0.6) | (0.0) |
| Urban household | 0.044 | 0.010 | 0.020 | 0.052 | 0.019 | -0.003 |
|  | (0.2) | (0.0) | (0.4) | (0.0) | (0.3) | (0.0) |
| Hindu household | -0.046 | 0.003 | 0.242 | -0.060 | -0.219 | 0.029 |
|  | (0.2) | (0.0) | (0.2) | (0.0) | (0.3) | (0.0) |
| Muslim household | -0.514 | 0.056 | -0.044 | 0.031 | -0.757 | 0.077+ |
|  | (0.4) | (0.0) | (0.4) | (0.1) | (0.5) | (0.0) |
| Scheduled caste | -0.038 | 0.008 | -0.280 | 0.040 | 0.024 | -0.002 |
|  | (0.2) | (0.0) | (0.2) | (0.0) | (0.2) | (0.0) |
| Scheduled tribe | -0.792* | 0.123** | -0.315 | 0.132 | -0.989* | 0.127* |
|  | (0.3) | (0.0) | (0.6) | (0.1) | (0.4) | (0.0) |
| Other backward classes | 0.098 | -0.010 | 0.367 | 0.015 | 0.039 | -0.020 |
|  | (0.3) | (0.0) | (0.3) | (0.0) | (0.3) | (0.0) |
| Proportion of households openly defecating in village | 0.252 | 0.031 | 0.073 | 0.007 | -0.112 | 0.055 |
|  | (0.8) | (0.0) | (0.4) | (0.0) | (0.9) | (0.1) |
| Constant | 7.946*** | 1.000*** | 6.849*** | 1.068*** | 8.795*** | 0.951*** |
|  | (0.8) | (0.1) | (0.7) | (0.1) | (1.0) | (0.1) |
| R^2^ | 0.028 | 0.121 | 0.180 | 0.234 | 0.021 | 0.106 |
| N | 3608 | 3608 | 842 | 842 | 2766 | 2766 |
| + p<0.10, * p<0.05, ** p<0.01, *** p<0.001  Standard errors in parentheses have been corrected for clustering at the district level.  ^a^Anaemia defined as hemoglobin < 110 g/L per World Health Organization criteria for pregnant women [3].  Hb, haemoglobin; HR, Haryana; PN, Punjab | | | | | | |

| **Supplementary Table 3. Difference-in-difference estimate of fortifying wheat through the PDS on anemia - Tamil Nadu vs Andhra Pradesh, Kerala and Karnataka** | | | | | | |
| --- | --- | --- | --- | --- | --- | --- |
|  | **TN vs AP, KR, KA** | | **TN vs AP, KR, KA – bordering districts** | | **TN vs AP, KR, KA – non-bordering districts** | |
| **Model 🡪** | **(1)** | **(2)** | **(3)** | **(4)** | **(5)** | **(6)** |
| **Predictor Outcome 🡪** | **Hb** | **Anemia^a^** | **Hb** | **Anemia^a^** | **Hb** | **Anemia^a^** |
| Diff-in-diff estimator (treatment dummy*post treatment year) | -0.001 | -0.075* | -1.298 | 0.008 | 0.502 | -0.090* |
|  | (0.5) | (0.0) | (1.4) | (0.1) | (0.4) | (0.0) |
| Treatment dummy (=1 for Tamil Nadu) | 0.504 | -0.023 | 1.629 | -0.103 | -0.061 | 0.024 |
|  | (0.5) | (0.0) | (1.1) | (0.1) | (0.5) | (0.0) |
| Post treatment year (=1 if year is 2012-13) | -0.019 | -0.172*** | 0.199 | -0.202* | -0.063 | -0.166*** |
|  | (0.3) | (0.0) | (0.9) | (0.1) | (0.3) | (0.0) |
| Woman's years of education | 0.032* | -0.001 | 0.060+ | -0.002 | 0.019 | 0.000 |
|  | (0.0) | (0.0) | (0.0) | (0.0) | (0.0) | (0.0) |
| Woman's age | 0.038** | -0.004*** | 0.075** | -0.005+ | 0.024+ | -0.003** |
|  | (0.0) | (0.0) | (0.0) | (0.0) | (0.0) | (0.0) |
| Number of IFA tablets consumed by woman | -0.001+ | 0.000 | -0.003+ | 0.000** | -0.001 | -0.000 |
|  | (0.0) | (0.0) | (0.0) | (0.0) | (0.0) | (0.0) |
| Household cooking fuel is wood | 0.252 | -0.000 | 0.628 | -0.043 | 0.115 | 0.015 |
|  | (0.2) | (0.0) | (0.5) | (0.0) | (0.2) | (0.0) |
| Woman was married before 18 years of age | -0.119 | -0.007 | 0.141 | -0.021 | -0.145 | -0.003 |
|  | (0.1) | (0.0) | (0.3) | (0.0) | (0.1) | (0.0) |
| Lowest wealth quintile (base category) | 0.000 | 0.000 | 0.000 | 0.000 | 0.000 | 0.000 |
|  | (.) | (.) | (.) | (.) | (.) | (.) |
| Second lowest wealth quintile | 0.521* | -0.023 | 0.361 | -0.004 | 0.559** | -0.028+ |
|  | (0.2) | (0.0) | (0.6) | (0.0) | (0.2) | (0.0) |
| Middle wealth quintile | 0.203 | 0.006 | -0.344 | 0.023 | 0.335 | 0.002 |
|  | (0.2) | (0.0) | (0.5) | (0.0) | (0.2) | (0.0) |
| Second highest wealth quintile | 0.147 | -0.012 | -0.357 | 0.031 | 0.278 | -0.025 |
|  | (0.2) | (0.0) | (0.5) | (0.0) | (0.2) | (0.0) |
| Highest wealth quintile | 0.365 | -0.043 | 0.413 | -0.080 | 0.329 | -0.027 |
|  | (0.3) | (0.0) | (0.7) | (0.1) | (0.3) | (0.0) |
| Urban household | 0.161 | -0.028 | 0.021 | 0.003 | 0.218 | -0.044* |
|  | (0.2) | (0.0) | (0.4) | (0.0) | (0.2) | (0.0) |
| Hindu household | -0.617+ | 0.015 | -0.497 | 0.024 | -0.334 | -0.028 |
|  | (0.3) | (0.0) | (0.4) | (0.0) | (0.4) | (0.0) |
| Muslim household | -0.469 | -0.007 | -0.796 | 0.009 | -0.032 | -0.050 |
|  | (0.4) | (0.0) | (0.5) | (0.0) | (0.4) | (0.0) |
| Scheduled caste | -0.084 | -0.012 | -1.154** | 0.077+ | 0.256 | -0.042* |
|  | (0.2) | (0.0) | (0.3) | (0.0) | (0.2) | (0.0) |
| Scheduled tribe | -0.417+ | 0.010 | -0.765 | 0.037 | -0.327 | 0.002 |
|  | (0.2) | (0.0) | (0.5) | (0.1) | (0.3) | (0.0) |
| Other backward classes | 0.149 | -0.020 | -0.043 | 0.029 | 0.166 | -0.032+ |
|  | (0.2) | (0.0) | (0.3) | (0.0) | (0.2) | (0.0) |
| Proportion of households openly defecating in village | -0.070 | -0.003 | 0.400 | 0.053 | -0.087 | -0.030 |
|  | (0.4) | (0.0) | (1.0) | (0.1) | (0.4) | (0.0) |
| Constant | 8.908*** | 1.001*** | 8.321*** | 0.910*** | 8.898*** | 1.058*** |
|  | (0.7) | (0.1) | (1.3) | (0.1) | (0.8) | (0.1) |
| R^2^ | 0.030 | 0.086 | 0.050 | 0.096 | 0.024 | 0.082 |
| N | 6578 | 6578 | 1574 | 1574 | 5004 | 5004 |
| + p<0.10, * p<0.05, ** p<0.01, *** p<0.001  Standard errors in parentheses have been corrected for clustering at the district level.  ^a^Anaemia defined as hemoglobin < 110 g/L per World Health Organization criteria for pregnant women [3].  AP, Andhra Pradesh; Hb, haemoglobin; KA, Karnataka; KR, Kerala; PDS, public distribution system; TN, Tamil Nadu | | | | | | |

| **Supplementary Table 4. Triple-difference estimates - Punjab versus Haryana** | | | | | | |
| --- | --- | --- | --- | --- | --- | --- |
|  | **PN vs HR** | | **PN vs HR - bordering districts** | | **PN vs HR - non-bordering districts** | |
| **Model 🡪** | **(1)** | **(2)** | **(3)** | **(4)** | **(5)** | **(6)** |
| **Predictor Outcome 🡪** | **Hb** | **Anaemia^a^** | **Hb** | **Anaemia^a^** | **Hb** | **Anaemia^a^** |
| Triple difference estimator (treatment dummy*post treatment year*APL) | 0.536 | -0.079 | 0.805 | -0.105 | 0.395 | -0.052 |
|  | (0.8) | (0.1) | (1.3) | (0.2) | (1.0) | (0.1) |
| APL*treat | 0.289 | -0.021 | 0.182 | 0.104 | 0.284 | -0.059 |
|  | (0.7) | (0.0) | (0.6) | (0.1) | (0.9) | (0.0) |
| APL*post treatment time dummy | 0.618 | -0.004 | 0.574 | -0.010 | 0.649 | -0.015 |
|  | (0.4) | (0.0) | (0.7) | (0.1) | (0.4) | (0.0) |
| Diff-in-diff estimator (treatment dummy*post treatment year) | -0.826 | 0.074 | -0.176 | 0.144 | -0.963 | 0.043 |
|  | (0.8) | (0.1) | (0.6) | (0.1) | (1.0) | (0.1) |
| Treatment dummy (=1 for Punjab) | 0.281 | 0.002 | -0.795+ | -0.029 | 0.553 | 0.012 |
|  | (0.7) | (0.0) | (0.4) | (0.0) | (0.9) | (0.1) |
| Post treatment year (=1 if year is 2012-13) | 0.613 | -0.262*** | 1.118** | -0.357** | 0.341 | -0.225*** |
|  | (0.5) | (0.1) | (0.3) | (0.1) | (0.5) | (0.1) |
| Dummy for being APL | -0.581 | 0.065* | -0.589 | -0.023 | -0.611 | 0.101*** |
|  | (0.4) | (0.0) | (0.5) | (0.1) | (0.5) | (0.0) |
| Woman's years of education | 0.042* | -0.007** | 0.097** | -0.011* | 0.015 | -0.005* |
|  | (0.0) | (0.0) | (0.0) | (0.0) | (0.0) | (0.0) |
| Woman's age | 0.035* | -0.004* | 0.035 | -0.003 | 0.029 | -0.004 |
|  | (0.0) | (0.0) | (0.0) | (0.0) | (0.0) | (0.0) |
| Number of IFA tablets consumed by woman | 0.003 | 0.000 | 0.001 | 0.000 | 0.003 | 0.000 |
|  | (0.0) | (0.0) | (0.0) | (0.0) | (0.0) | (0.0) |
| Household cooking fuel is wood | 0.258+ | -0.008 | 0.118 | -0.021 | 0.348+ | -0.006 |
|  | (0.1) | (0.0) | (0.2) | (0.0) | (0.2) | (0.0) |
| Woman was married before 18 years of age | -0.415* | 0.011 | 0.030 | -0.034 | -0.546** | 0.025 |
|  | (0.2) | (0.0) | (0.2) | (0.0) | (0.2) | (0.0) |
| Urban household | 0.078 | 0.008 | 0.075 | 0.049 | 0.051 | -0.005 |
|  | (0.2) | (0.0) | (0.4) | (0.0) | (0.3) | (0.0) |
| Hindu household | -0.035 | 0.002 | 0.192 | -0.067 | -0.202 | 0.027 |
|  | (0.2) | (0.0) | (0.2) | (0.0) | (0.3) | (0.0) |
| Muslim household | -0.479 | 0.055 | 0.120 | -0.003 | -0.711 | 0.081* |
|  | (0.4) | (0.0) | (0.4) | (0.1) | (0.5) | (0.0) |
| Scheduled caste | -0.084 | 0.012 | -0.334 | 0.056 | -0.015 | -0.000 |
|  | (0.2) | (0.0) | (0.2) | (0.0) | (0.3) | (0.0) |
| Scheduled tribe | -0.810** | 0.126** | -0.435 | 0.146 | -0.980* | 0.127* |
|  | (0.3) | (0.0) | (0.6) | (0.1) | (0.4) | (0.0) |
| Other backward classes | 0.061 | -0.007 | 0.310 | 0.029 | 0.010 | -0.018 |
|  | (0.3) | (0.0) | (0.3) | (0.0) | (0.3) | (0.0) |
| Proportion of households openly defecating in village | 0.152 | 0.042 | -0.105 | 0.021 | -0.171 | 0.063 |
|  | (0.7) | (0.0) | (0.4) | (0.0) | (0.9) | (0.0) |
| Constant | 8.086*** | 0.987*** | 7.482*** | 1.085*** | 8.819*** | 0.922*** |
|  | (0.7) | (0.1) | (0.8) | (0.1) | (0.9) | (0.1) |
| R^2^ | 0.028 | 0.121 | 0.178 | 0.231 | 0.022 | 0.107 |
| N | 3608 | 3608 | 842 | 842 | 2766 | 2766 |
| ^a^Anaemia defined as hemoglobin < 110 g/L per World Health Organization criteria for pregnant women [3]  + p<0.10, * p<0.05, ** p<0.01, *** p<0.001  Data for predictors are presented at beta coefficients with standard errors in parentheses  Standard errors in parentheses have been corrected for clustering at the district level.  APL, above poverty line; Hb, haemoglobin; HR, Haryana; PDS, public distribution system; PN, Punjab | | | | | | |
